# Synthesis and Structure of New 3,3,9,9-Tetrasubstituted-2,4,8,10-Tetraoxaspiro[5.5]undecane Derivatives

**DOI:** 10.3390/molecules13112848

**Published:** 2008-11-17

**Authors:** Alin Mihiş, Eric Condamine, Elena Bogdan, Anamaria Terec, Tibor Kurtán, Ion Grosu

**Affiliations:** 1 Organic Chemistry Department and CCOCCAN, Babes-Bolyai University, Cluj-Napoca, 11 Arany Janos str., 400028, Cluj-Napoca, Romania; E-mail: mihis_a@yahoo.com (A. M.), ebogdan@chem.ubbcluj.ro (E. B.), asuciu@chem.ubbcluj.ro (A. T); 2 IRCOF, UMR 6014, Université de Rouen, 76821 Mont Saint-Aignan, Cedex, France. E-mail: eric.condamine@univ-rouen.fr (E. C.); 3 Organic Chemistry Department, University of Debrecen, P.O. Box 20, 4010 Debrecen, Hungary E-mail: kurtant@tigris.unideb.hu (T. K.)

**Keywords:** Spiranes, 1,3-Dioxanes, Axial chirality, Conformational analysis, Dynamic NMR.

## Abstract

The configurational and conformational behavior of some new 3,3,9,9-tetra-substituted-2,4,8,10-tetraoxaspiro[5.5]undecane derivatives with axial chirality was investigated by conformational analysis and variable temperature NMR experiments.

## Introduction

**Scheme 1:**
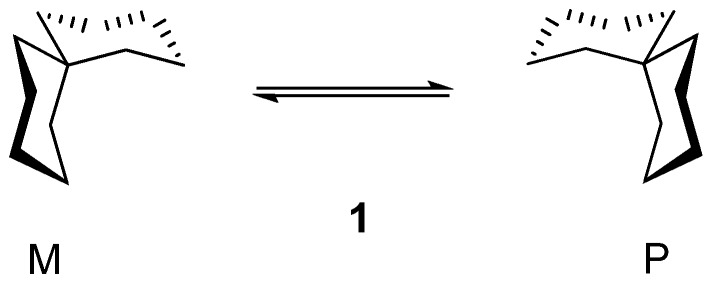
Helical structure of spiro[5.5]undecane (**1**).

The stereochemistry of spiranes with six-membered rings has been extensively studied [1]. Most of these investigations were carried out with spiranes containing 1,3-dioxane units [1,2,3,4,5,6,7,8,9,10]. The chirality of the parent spiro[5.5]undecane (**1**, Scheme 1) was observed by Dodziuk [3,4], but at that time no chiral element according to the classification of Cahn, Ingold and Prelog [11,12] was found and the chirality of the spiro compounds with six-membered rings could not be satisfactorily explained. Later, we observed the possibility of the helical arrangement in polyspiranes with six-membered rings [5,6]. In these polyspiranes the helix can become identical with itself after each fourth six-membered ring. In monospiranes (*e.g.* compound **1**) the helix begins to be built and the two enantiomeric structures exhibit either *P* or *M* configuration (Scheme 1).

**Scheme 2:**
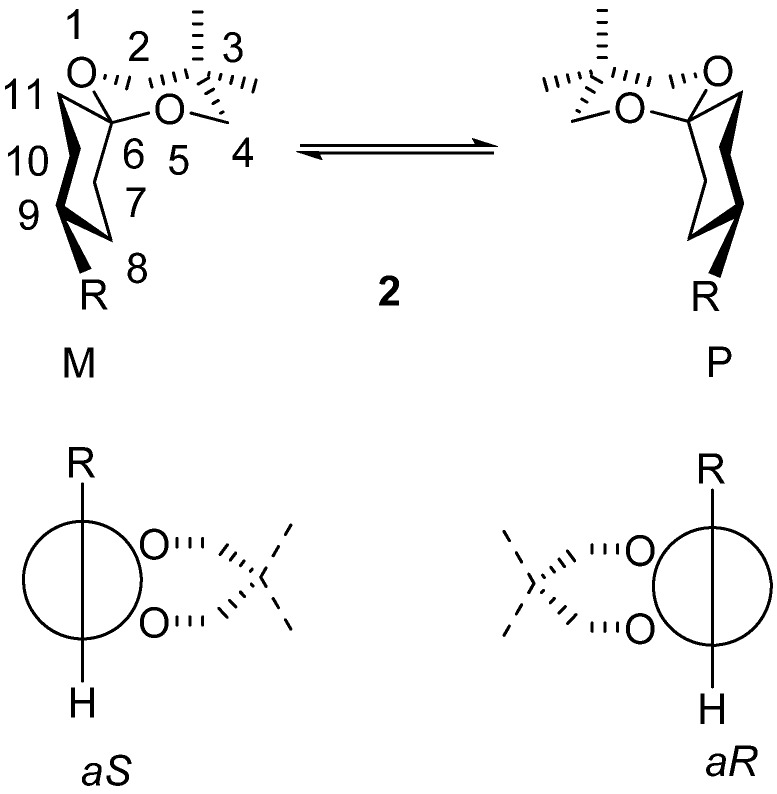
Axial chirality of semiflexible spiranes with 1,5-dioxaspiro[5.5]undecane skeleton.

On the other hand the axial chirality of these spiranes is also different. For instance, the semiflexible derivatives **2** of 1,5-dioxaspiro[5.5]undecane (Scheme 2) with helical chirality (as a result of the specific arrangement of the spirane skeleton) exhibit axial chirality too, despite the similar substituents located at one of the ends (position 3) of the spirane [5,6] system.

The C^6^-C^9^ axis is a chiral element and the different groups at the ends of this axis are R and H at C^9^ and the 1,3-dioxane ring on one side and the missing ligand on the other side at C^6^. In these compounds (**2**) the carbocycle is anancomeric and the heterocycle is flipping. This conformational equilibrium (flipping of the heterocycle) is an enantiomeric inversion (Scheme 2).

**Scheme 3:**
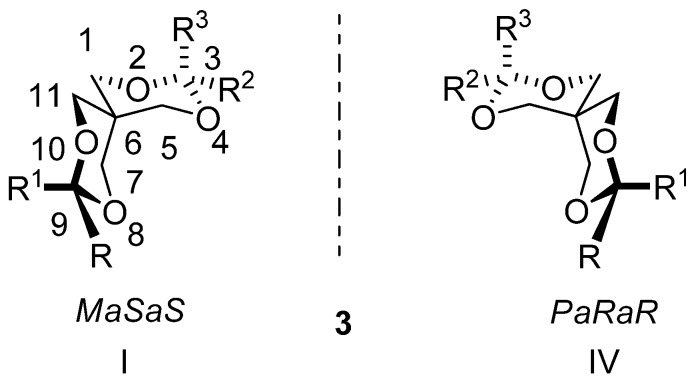
Main conformers of tetrasubstituted anancomeric spiranes with 2,4,8,10-tetra-oxaspiro[5.5]undecane skeleton.

Compounds with 2,4,8,10-tetraoxaspiro[5.5]undecane skeleton (**3**, Scheme 3) bearing different substituents at both ends of the spirane system were also investigated. In these compounds, besides the helical chirality, two chiral axis (C^3^-C^6^ and C^6^-C^9^) can be considered and six isomers are possible (Table 1) [6,13]. In all studied compounds **3** there are large conformational energy differences between the substituents located at the same positions and the compounds exhibit anancomeric structures. If substituents R and R^2^ have a considerably higher free energy than the other substituents located at the same positions (R^1^ and R^3^) the preferred structures (I and IV) exhibit these groups in equatorial orientations. Structures I and IV are considered representative for compounds **3**, they cannot be transformed into one other by conformational processes and thus represent separable enantiomers.

**Table 1:** Possible stereoisomers for the monospirane with two chiral axes and helical chirality.

Isomer	Configuration of the helix	Configuration of chiral axes		Orientation of the bulky substituents	
		C^3^-C^6^	C^6^-C^9^	C^3^ (R)	C^9^ (R^2^)
I	*M*	*aS*	*aS*	eq	eq
II	*P*	*aR*	*aS*	ax	eq
II’*	*P*	*aS*	*aR*	eq	ax
III	*M*	*aR*	*aR*	ax	ax
IV	*P*	*aR*	*aR*	eq	eq
V	*M*	*aS*	*aR*	ax	eq
V’*	*M*	*aR*	*aS*	eq	ax
VI	*P*	*aS*	*aS*	ax	ax

*Because similar substitutions at C^3^ and C^9^ structures II and II’; V and V’ are equivalent.

We considered of interest to investigate the stereochemistry (conformational analysis and enantiomerism) of 3,3,9,9-tetrasubstituted-2,4,8,10-tetraoxaspiro[5.5]undecane derivatives with different substituents at the same position (similar to compounds **3**) which exhibit flexible structures. 

## Results and Discussion

**Scheme 4:**
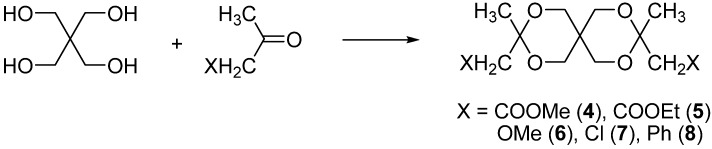
Synthesis of spiranes **4**-**8**.

New spiro compounds with 2,4,8,10-tetraoxaspiro[5.5]undecane skeleton **4**-**8** were obtained by the condensation of pentaerythritol with non-symmetrical ketones (Scheme 4).

**Scheme 5:**
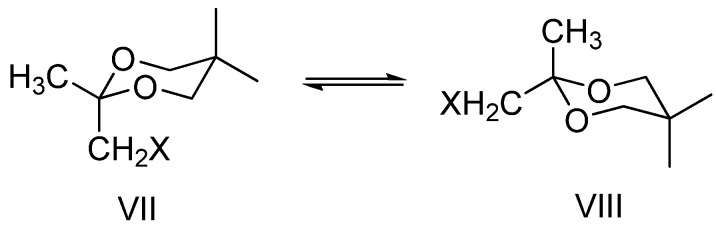
Conformational equilibria for 2,2-disubstituted-1,3-dioxane derivatives.

In previous studies [14,15] it was demonstrated that CH_3_ and CH_2_X groups located in the ketal part of the 1,3-dioxane ring (position 2) have very close conformational free enthalpies and 2-CH_3_,2-CH_2_X-1,3-dioxane derivatives are flexible compounds (Scheme 5) and conformers VII and VIII have similar contributions to the average structure of the compound.

**Scheme 6:**
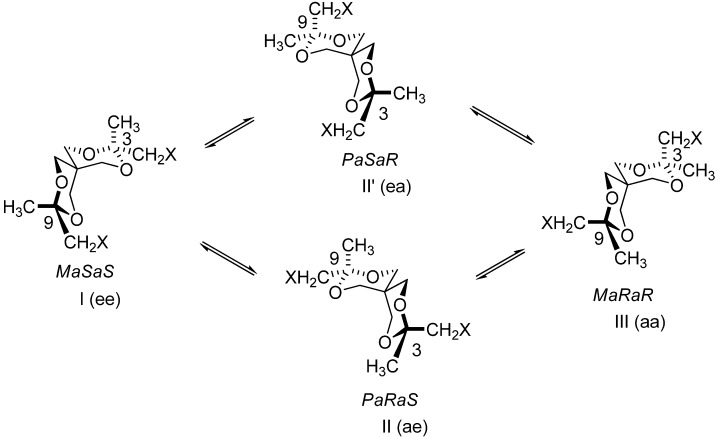
Conformational equilibria involving diastereomeric structures I-III.

**Scheme 7:**
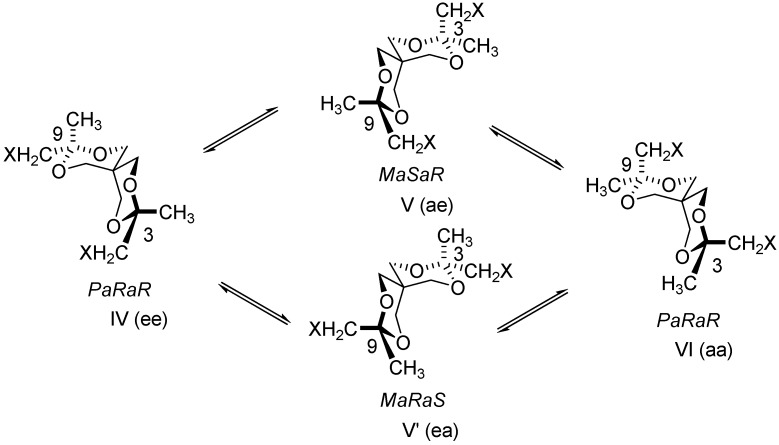
Conformational equilibria involving diastereomeric structures IV-VI.

Taking into account these data compounds **4**-**8** were considered flexible. They exhibit (like compounds **3**) six conformers (Table 1). These conformers form two groups [I, II (II’), III and IV, V (V’), VI] involved in the equilibria I⇆II(II’)⇆III and IV⇆V(V’)⇆VI (Scheme 6 and Scheme 7). 

**Figure 1:**
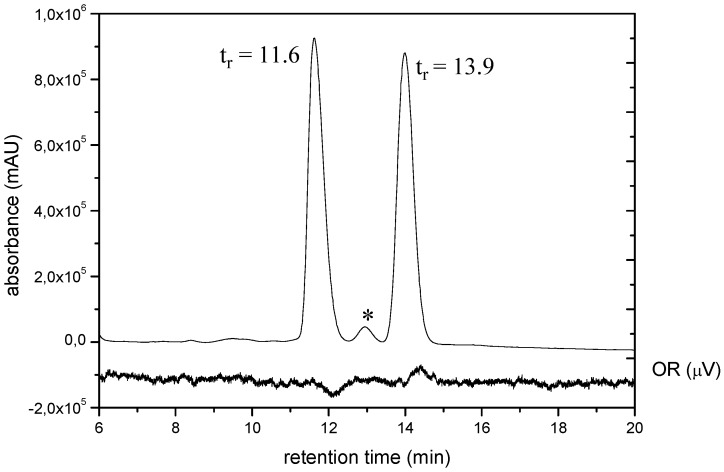
HPLC chromatograms of **8** on Chiralcel OD column using UV (211 nm) and OR detection.

The conformers of each group are diastereoisomers [ee, ae(ea), aa; CH_2_X is taken as reference] and they have an enantiomer in the other group. To transform a structure of one group into a structure of the other group it is necessary to break bonds and to remake bonds. Compounds **4**-**8**, despite their flexible structure, exhibit separable enantiomers. In order to discriminate the enantiomers, chiral HPLC experiments, using a CHIRALCEL OD column and normal and chiral (OR) detections, were run with compounds **5** and **8**. The peaks of the enantiomers are baseline separated (*t*_r_s for **5**: 27.34, 33.05 min and for **8**: 11.6, 13.9 min; Figure 1), but the signals in CD detection are weak probably because these ones are the average of similar contributions belonging to diastereoisomers (see scheme 6 and scheme 7) with opposite optical activity. 

The flexible structure of the compounds was revealed by dynamic ^1^H- and ^13^C-NMR experiments. The room temperature (*rt*) ^1^H-NMR spectrum exhibits for the three diastereoisomers of the compounds only one set of signals at mean values of the chemical shifts. 

**Figure 2:**
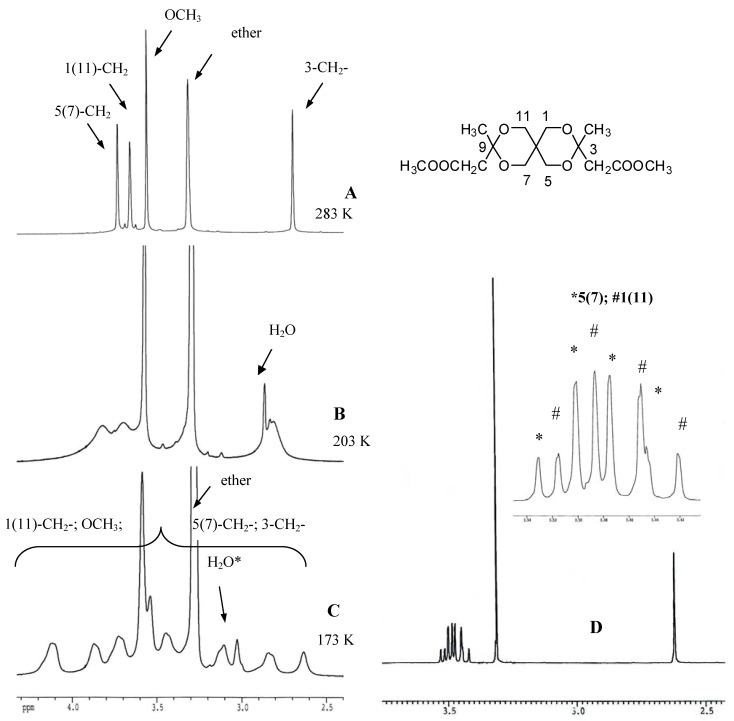
^1^H-NMR experiments run with compound **4** [diethylether-*d_10_*: 283 K (A), 203 K (B), 173 K (C); benzene-*d_6_*: *rt* (D)]; *The water signal is moving to higher δ values by diminishing the temperature.

For instance the spectrum of **4** run in Et_2_O-*d_10_* at 283 K (Figure 2, A) shows unique signals (singlets) for the protons of the heterocycles [δ_1(11)_ = 3.68 (the beginning of an AB splitting is observed) and δ_5(7)_ = 3.75 ppm] and for those of the substituents located at positions 3 and 9 [δ(OCH_3_) = 3.59, δ(CH_2_) = 2.73 and δ(CH_3_) = 1.08 ppm]. Despite the flexible structure of the compound, positions 1,11 and 5,7 are diastereotopic and give different signals, so they are not rendered equivalent by conformational equilibria. The *rt* spectrum of **4** run in C_6_D_6_ reveals the diastereotopicity of protons located at the same position (one is *procis* and the other one is *protrans* referred to the substituent with higher precedence located at the closer extremity of the spirane skeleton) so in this case the pattern of the spectrum for the protons of the spirane skeleton consists of two AB systems (Figure 2, D). 

The variable temperature ^1^H-NMR experiment run with **4** reveals the coalescence of the signals (T = 203 K) and at lower temperature separated groups of signals which correspond to the three frozen diastereoisomers and to the axial and equatorial positions of the protons of the spirane and of the groups located at it. The complete assignment of the signals in the low temperature spectrum was not possible, but the evolution of the pattern of the spectra observed by diminishing the temperature clearly shows the freezing of conformational equilibria and the obtaining at low temperature of frozen diastereoisomers in agreement with the structures shown in schemes 6 and 7. The results obtained in the variable temperature ^1^H-NMR experiments run with the other compounds of this series are similar with those shown for **4**.

**Figure 3:**
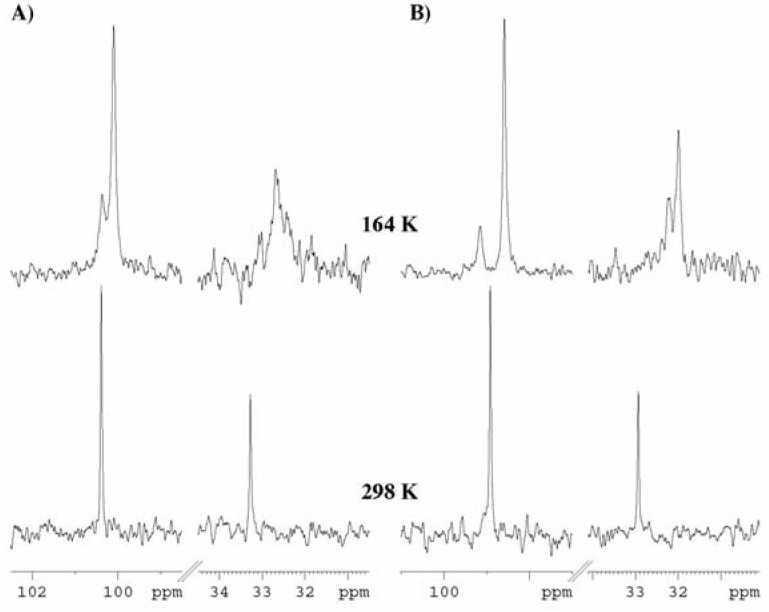
Fragments [C^3(9)^ and C^6^] of the ^13^C-NMR spectra of compounds **8** (A) and **4** (B) recorded at 298 K and 164 K.

Variable temperature ^13^C-NMR experiments (300 – 164 K) were run with compounds **4**, **6** and **8** in THF-*d8* (Table 2, Figure 3). The coalescence of the signals for **6** was observed at lower temperature (T_c_=170 K) in comparison with the results in the ^1^H-NMR variable temperature experiment run with the same compound in the same conditions. 

**Table 2:** Results (δ, ppm) of the variable temperature ^13^C NMR experiments run with compounds **4** and **8**. ^#^

Compd	C^3^, C^9^		C^1^, C^11^; C^5^, C^7^		C^6^		CO/Car	
	*rt*	164 K	*rt*	164 K*	*rt*	164 K	*rt*	164 K*
**4**	98.92	99.15 98.59	64.47 64.42	63.22 63.60 63.87	32.93	31.99 32.20	170.03	170.43 170.72
**8**	100.39	100.09 100.37	64.49 64.54	63.55 63.81	33.28	–	(a) 128.40 (b) 138.13	(a) 128.51 128.87 (b) 137.70 138.89

* These signals belong to C^1^, C^11^ the other group of signals belonging to C^5^, C^7^ are overlapped at 164 K with the signals of the solvent. # Some of the signals are still in coalescence at 164 K.

For **4** and **8** the coalescences in the ^13^C-NMR take place at similar or higher temperatures than in ^1^H-NMR. At low temperature (164 K) the ^13^C-NMR spectra (Table 2, Figure 3) are more complicated and exhibit many signals suggesting the freezing of the conformational equilibria - instead of each signal recorded at *rt* two or more signals (with different intensities) belonging to the frozen diastereoisomers appear. 

Despite these favorable results the assignment of the signals to each of the frozen diastereoisomers of **4** and **8** was not possible. However, these results prove that at *rt* conformational equilibria between many distereoisomers (three according to Scheme 6 and Scheme 7) are present. Attempted determinations of X-ray crystal structures by diffraction for **7** and **8** failed. This was considered to be a consequence of the fact that in solid state the compounds are mixtures of all possible diastereoisomers, whose resolution was not possible. The X-ray crystal structures obtained for similar compounds [16] reveal the preference of the six-membered rings for the chair conformation in solid state, too.

## Conclusions

Spiro compounds **4**-**8** bearing groups with similar conformational enthalpies at the ends of the spirane skeleton show flexible structures. The compounds exhibit separable enantiomers and for each enantiomer the flipping of the six membered rings equilibrate three diastereoisomers which are not separable. The flexible structure of the compounds was revealed by variable temperature ^1^H and ^13^C NMR experiments and the chiral behavior of the molecules was proved by chiral HPLC discriminations.

## Experimental

### General

Routine ^1^H-NMR (300 MHz) and ^13^C-NMR (75 MHz) spectra were recorded at room temperature in CDCl_3_ on a Bruker 300 MHz spectrometer, using the solvent line as reference. Variable temperature NMR spectra were recorded on Bruker Avance DMX 500 spectrometer in CD_2_Cl_2_, (CD_2_)_4_O or (C_2_D_5_)_2_O. Electron impact (70 eV) mass spectra were obtained on Hewlett-Packard MD 5972 GC-MS instrument. GC analyses were performed on a Hewlett-Packard HP 5890 gas chromatograph. A HP-5MS capillary column (30 m x 0.25 mm x 0.33 µm) and helium gas were used for separations.Electrospray ionisation mass spectra ESI (ESI^+^) were recorded using an Esquire 3000 ion-trap mass spectrometer (Bruker Daltonic GmbH, Bremen, Germany) equipped with a standard ESI/APCI source. HPLC separations were carried out with a Jasco HPLC system on Chiralcel OD column (5 mm, 250 x 4.6 mm equipped with a 50 x 4.6 mm OD guard column) termostated at 25 °C with eluent hexane : isopropanol 9:1 and 1 ml/min flow rate. A JASCO MD-910 multiwavelength detector and a JASCO OR-2090 chiral detector were used for detection. Melting points were measured with a Kleinfeld melting point apparatus and are uncorrected. Thin layer chromatography (TLC) was conducted on silica gel 60 F254 TLC plates purchased from Merck. Preparative column chromatography was performed using PharmPrep 60 CC (40–63 µm) silica gel purchased from Merck. Chemicals were purchased from Aldrich, Merck or Acros and were used without further purification.

### General procedure for preparation of **4**-**8**

A mixture of ketone (48 mmol) and pentaerythritol (20 mmol) and catalytic amounts of *p*-toluene-sulfonic acid (0.1 g) was dissolved in 100−150 mL benzene (compounds **4**−**6** and **8**) or toluene (compound **7**). The reaction mixture was refluxed and the water was removed by azeotropic distillation using a Dean-Stark trap. When 90 % of the theoretical amount of water had been separated, the mixture was cooled to room temperature and the catalyst was neutralized (under stirring over 1 h) with sodium acetate powder in excess (0.3 g). The reaction mixture was washed with water (2 x 100 mL). After drying with anhydrous sodium sulfate, the solvent was removed *in vacuo* and the crude product was purified by distillation, column chromatography or by crystallization from methanol.

*3,9-Dimethyl-3,9-bis(methoxycarbonylmethyl)-2,4,8,10-tetraoxaspiro[5.5]undecane* (**4**). Yield: 57%; colorless oil; *R_f_* = 0.22 (*n*-hexane / ethyl acetate = 8:2); ^1^H-NMR: δ/ppm 1.52 [s, 6H, 3(9)-CH_3_], 2.76 [s, 4H, 3(9)-CH_2_-CO-], 3.69 [s, 6H, 3(9)-CH_2_-COO*CH*_3_], 3.71-3.81 [overlapped peaks, 8H, 1(11)-CH_2_-, 5(7)-CH_2_-];^13^C-NMR: δ/ppm 21.5 [3(9)-CH3], 32.4 (C^6^), 42.1 [3(9)-CH2-], 51.7 [3(9)-CH2COO*CH*_3_], 63.8 (C^1^, C^5^, C^7^, C^11^), 98.1 (C^3^, C^9^), 169.6 [3(9)-COO-]; MS (EI, 70 eV) m/z (%): 317 (16), 259 (69), 143 (14), 117 (31), 101 (22), 83 (50), 43 (100); Anal. Calcd for C_15_H_24_O_8_ (332.15): C, 54.21; H, 7.28; found: C, 54.47; H, 7.09.

*3,9-bis(Ethoxycarbonylmethyl)-3,9-dimethyl-2,4,8,10-tetraoxaspiro[5.5]undecane* (**5**). Yield: 35%; colorless oil; bp 207 ºC/2 mm; ^1^H-NMR: δ/ppm 1.25 [t, 6H, *^3^J* = 7.1 Hz, 3(9)-COO-CH_2_C*H*_3_], 1.52 [s, 6H, 3(9)-CH_3_], 2.75 [s, 4H, 3(9)-CH_2_-CO], 3,77 (overlapped peaks, 8H, 1-CH_2_, 5-CH_2_, 7-CH_2_, 11-CH_2_), 4.14 [q, 4H, *^3^J* = 7.1 Hz, 3(9)-CH_2_COO-C*H*_2_CH_3_]; ^13^C-NMR: δ/ppm 14.0 [3(9)-CH_2_-COO-CH_2_*C*H_3_] 22.0 [3(9)-CH_3_], 32.2 (C^6^), 41.56 [3(9)-CH_2_-], 60.4 [3(9)-CH_2_-COO*CH_2_*-], 63.7 (C^1^, C^5^, C^7^, C^11^), 98.0 (C^3^, C^9^), 169.1 [3(9)-COO-]; MS (ESI): 383.16 [M + Na^+^]; MS (EI, 70 eV) m/z (%): 345 (8), 273 (51), 203 (6), 185 (10), 143 (12), 131 (25), 113 (21), 83 (41), 43 (100); Anal. Calcd for C_17_H_28_O_8_ (360.18): C, 56.65; H, 7.83; found: C, 56.38; H, 7.65.

*3,9-Dibenzyl-3,9-dimethyl-2,4,8,10-tetraoxaspiro[5.5]undecane* (**6**). Yield: 29%; white solid; mp 97−98 ˚C; *R_f_* = 0.16 (*n*-hexane); ^1^H-NMR: δ/ppm 1.29 [s, 6H, 3(9)-CH_3_], 2.98 [s, 4H, 3(9)-CH_2_-], 3.63 [d, 2H, *^2^J* = 11.7 Hz, 1(11)-H], 3.72 [d, 2H, *^2^J* = 11.7 Hz, 1(11)-H’], 3.73 [d, 2H, *^2^J* = 11.7 Hz, 5(7)-H], 3.86 [d, 2H, *^2^J* = 11.7 Hz, 5(7)-H’], 7.23−7.31 [overlapped peaks, 10H, 3(9)-CH_2_-C_6_*H*_5_]; ^13^C-NMR: δ/ppm 19.7 [3(9)-CH_3_], 33.0 (C^6^), 44.4 [3(9)-CH_2_-], 63.9 (C^1^, C^11^), 64.0 (C^5^, C^7^), 99.9 (C^3^, C^9^), 126.4, 127.9, 130.6 (tertiary aromatic carbon atoms), 136.6 (quaternary aromatic carbon atom); MS (EI, 70 eV) m/z (%): 277 (80), 143 (65), 113 (31), 91 (40), 83 (65), 43 (100); Anal. Calcd for C_23_H_28_O_4_ (368.20): C, 74.97; H, 7.66; found: C, 75.20; H, 7.39.

*3,9-bis(Methoxymethyl)-3,9-dimethyl-2,4,8,10-tetraoxaspiro[5.5]undecane* (**7**). Yield: 36%; white solid; mp 66−67 ºC; *R_f_* = 0.40 (dichloromethane / ethyl acetate = 2:1); ^1^H-NMR: δ/ppm 1.41 [s, 6H, 3(9)-CH_3_], 3.37-3.39 [overlapped peaks, 4H, 1(11)-CH_2_], 3.40 [s, 6H, 3(9)-CH_2_OC*H*_3_], 3.64 [s, 4H, 3(9)-CH_2_-O-], 3.80 [d, 2H, *^2^J* = 11.8 Hz, 5(7)-H], 3.99 [d, 2H, *^2^J* = 11.8 Hz, 5(7)-H’]; ^13^C-NMR: δ/ppm 17.3 [3(9)-CH_3_], 32.9 (C^6^), 59.4 [3(9)-CH_2_O*C*H_3_], 63.6 (C^1^, C^11^), 63.7 (C^5^, C^7^), 75.9 [3(9)-CH_2_O-], 98.5 (C^3^, C^9^); MS (ESI): 299.14 [M + Na^+^]; MS (EI, 70 eV) m/z (%): 261 (3), 231 (63), 143 (31), 113 (25), 83 (67), 43 (100); Anal. Calcd for C_13_H_24_O_6_ (276.16): C, 56.51; H, 8.75; found: C, 56.63; H, 8.98.

*3,9-bis(Chloromethyl)-3,9-dimethyl-2,4,8,10-tetraoxaspiro[5.5]undecane* (**8**). Yield: 58%; white solid; mp 120−121 ºC; crystallized from methanol; ^1^H-NMR: δ/ppm 1.47 [s, 6H, 3(9)-CH_3_], 3.56 [s, 4H, 1(11)-CH_2_], 3.69 [s, 4H, 3(9)-CH_2_-Cl], 3.81 [d, 2H, *^2^J* = 11.7 Hz, 5(7)-H], 3.91 [d, 2H, *^2^J* = 11.7 Hz, 5(7)-H’]; ^13^C-NMR: δ/ppm 18.7 [3(9)-CH_3_], 32.8 (C^6^), 47.2 [3(9)-CH_2_-Cl], 64.0 (C^1^, C^11^), 64.1 (C^5^, C^7^), 98.4 (C^3^, C^9^); MS (EI, 70 eV) m/z (%): 269 (11), 235 (77), 143 (22), 113 (26), 83 (73), 43 (100); Anal. Calcd for C_11_H_18_Cl_2_O_4_ (284.06): C, 46.33; H, 6.36; Cl, 24.86; found: C, 46.08; H, 6.64; Cl, 25.03.
